# Medical education abroad: experience and perceived learning effects of German medical students in an international cardiology elective

**DOI:** 10.3389/fmed.2025.1556761

**Published:** 2025-07-09

**Authors:** Christian Albert, Katrin Werwick, Marc Gottschalk, Anna Aschoff, Angelo Auricchio, Rüdiger C. Braun-Dullaeus, Philipp Stieger

**Affiliations:** ^1^University Clinic for Cardiology and Angiology, Otto-von-Guericke University Magdeburg, Magdeburg, Germany; ^2^Department of Nephrology, Central Clinic Bad Berka, Bad Berka, Germany; ^3^Department of Strategic and Corporate Development, University Clinic Magdeburg, Magdeburg, Germany; ^4^Division of Cardiology, Istituto Cardiocentro Ticino, Lugano, Switzerland

**Keywords:** medical education, study abroad, international elective, internship abroad, cardiology, internal medicine, medical specialization, learning experience

## Abstract

**Background:**

With the aim of increasing study abroad courses the University Clinic for Cardiology and Angiology at the Otto-von-Guericke University Magdeburg (Magdeburg, Germany) in collaboration with the Istituto Cardiocentro Ticino (Lugano, Switzerland), established an intensive time-delimited student course to provide cardiological teaching content and insight beyond the regular curriculum. An underlying blueprint with learning objectives was formulated in order to ensure the training outcome. Within the present study, students’ individual perceptions of training in Italian-speaking Switzerland are assessed.

**Methods:**

Three focus groups discussions with elective graduates were performed (*N* = 18 course participants). Discussions were recorded, transcribed, and anonymized.

We performed a qualitative content analysis in accordance with Mayring and thereby formed an inductive category system. Categorized themes were analyzed to describe students’ perceptions and evaluations of the elective subjects.

**Results:**

In total *N* = 17/18 (94.4%) of the elective students participated in the group discussions. In general, teaching practical clinical and patient centered issues with high degree of interaction between the students and lecturers were acknowledged by the students.

In the Lugano elective, differences in the organization of the different healthcare systems and hospitals were noted. To experience medicine not only in a foreign context but also with different languages (English/Italian) was challenging but without particular problems.

Interdisciplinary and interprofessional aspects were less experienced. Ultimately, students showed a high level of reflection regarding their learning and teaching experience and gained more motivation to continue studying cardiology and a strong tendency to consider cardiology as a future specialist discipline.

**Conclusion:**

During the course of medical education, intensive courses and teachers’ engagement in particular topics can increase a student’s enthusiasm for the discipline. Similarly, temporary international experiences may foster interest in a selected area or discipline. Efforts to increase the possibilities in the medical school curriculum for studies abroad might positively influence the educational experience. Consideration may be given to implement such offerings at an earlier juncture to support interested students.

## Introduction

Curricular courses focusing on defined learning objectives (e.g., specialization seminars/elective subjects) have been successfully established at German medical schools. Such courses offer an additional teaching method to deepen core medical education, which is of particular value in inclination-oriented competence development (1). Structured subject-related teaching can improve the student’s experience and academic success and increase interest in a subject-specific career (2).

Many medical students spend individual internships, clinical traineeships, observerships, or study periods temporarily abroad (3). Although study abroad programs consider formal academic settings to be a crucial part of the program, participants mostly report learning from social and cultural situations, personal challenges, and field experiences as a reason for sojourning (4). In contrast to those courses encountered in familiar surroundings, everyday experiences that take place in a foreign setting with technical language can generate more endurable memories (5). However, while numerous studies have examined the general effects of single exchange experiences—such as increased self-efficacy—there is a lack of analysis on curricular study programs involving small groups and their potential impact on learning outcomes, students’ perception of the subject matter, and its influence on their choice of future specialization.

The effects of temporary study in a foreign environment are likewise assumed to be relevant regarding the subject and learning content, as well as an active merging of new knowledge with prior knowledge (6).

Specifically, for students interested in the field of cardiology, the effects of intensive, structured short stays at foreign institutions on their experience of the subject have not been sufficiently investigated. It remains unclear what influence intensive small group work in an international context has on the experience and the impression of cardiology in everyday clinical practice.

The aim of this study was therefore to investigate on students’ perceptions participating in an intensive and timely condensed cardiology elective in an international context at a specialty hospital in Lugano, Switzerland. Over the course of six semesters, students who participated in the elective were followed, and their motives, expectations and impressions were examined in focus group discussions and evaluated using qualitative content analysis. We hypothesize, that this approach may point out implications toward implementation of future educational models. We also hypothesize that this study will provide new insights into the perception and impact of internships abroad by medical students and that the subject-related learning achievements of international course participants will be clear. Curricular exchange programs, designed as international education initiatives in small groups, offer a promising approach to fostering the sustainability of learning objectives while enhancing students’ skills and nurturing their interest in future specialization areas. Finally, we anticipate that learning in foreign contexts may foster reflection and learning.

## Materials and methods

### Description of the cardiology elective

With the aim of increasing study abroad courses, the University Clinic for Cardiology and Angiology at Otto-von-Guericke University Magdeburg (Magdeburg, Germany) in collaboration with the Istituto Cardiocentro Ticino (Lugano, Italian-speaking Switzerland), established a course in 2015, in which up to six students of 3rd–5th year at medical school per each elective were able to voluntarily enroll in an intensive elective course in Lugano free of charge. Learning objectives for the cardiology elective were designed for a small group of students with an increased interest in the field of cardiology and was offered as intensive 8 h sessions for one-week at the Cardiocentro Ticino in Lugano, with sessions held in English.

The elective was structured as an intensive one-week program with daily sessions of approximately 8 h including joint breaks with educating physicians and colleagues for a coffee-time and a lunch in the staff-canteen. Around 60% of the course content consisted of theoretical instruction in the form of focused seminars, case-based discussions, and lectures provided by clinically active faculty members. Core topics included electrocardiography, rhythmology, heart failure, valvular heart disease, and cardiac surgery. Approximately 40% of the time was allocated to practical, clinical exposure, including ward-based teaching, supervised patient contact, and observation in diagnostic and interventional settings such as the cardiac catheterization laboratory and the intensive care unit. Students also took part in clinical conferences and morning meetings. Teaching was carried out in English and emphasized close interaction between students and instructors. Informal group reflection and peer discussion took place regularly after clinical sessions.

### Data collection through focus group discussion

Experiences and assessments of the participants of the seminar were clarified by focus group discussions of the following array of questions. The guideline (Supplementary Table 1) for the focus group discussion were developed *de novo* for this investigation, and included the following key points:

*Motivation and Expectations:*

• Students’ reasons for participating in the elective, their initial goals, and how their expectations shaped their engagement.

*Learning Experiences and Effects:*

• Didactic exposures during the elective, differences in the learning process compared to familiar environments, and the impact of intensive, subject-focused engagement in small groups within a foreign setting.

*Course Improvement Areas:*

• Identified areas for enhancing the elective experience based on student feedback.

*Potential Influence on Career Decisions:*

• How participation in the elective and subject-related immersion influences future career choices and further education plans.

*Peer Recommendations:*

• Whether students would recommend the elective subject to fellow students and the reasons for their endorsements.

**FIGURE 1:**
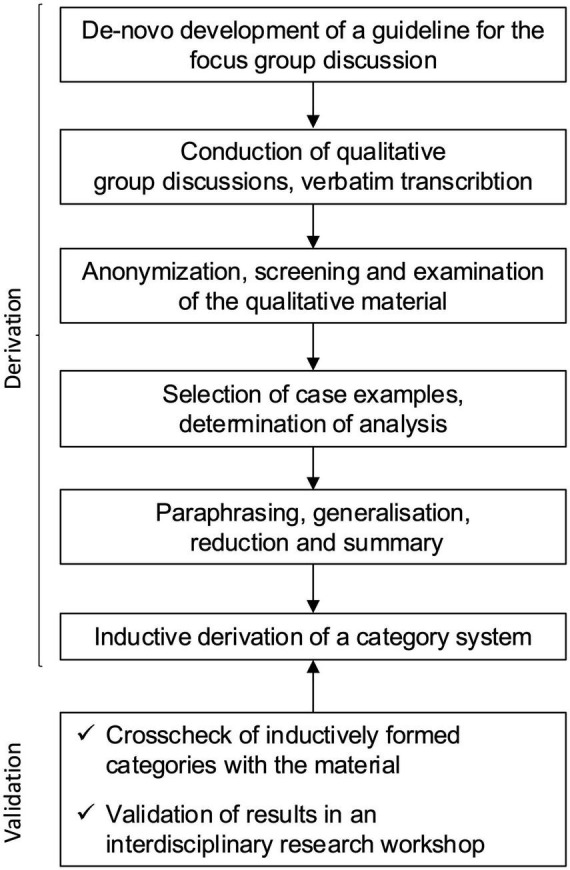
Study flow chart.

The focus group discussions were conducted immediately after returning from Lugano by trained research staff from our working group, who were not otherwise involved in the seminar program from 2015 to 2018, following participation in the elective. The study flowchart of the analysis approach is shown in Figure 1.

### Systematic approach to qualitative data analysis

The focus group discussions were performed in person by experienced interviewers, a medical doctor (AA) and a social scientist (KW), and subsequently analyzed using qualitative content analysis as proposed by Mayring (7), which involves a systematic procedure to ensure transparency and replicability of the findings from the material. Interviewers were not involved in the elective courses. No field notes were taken and there were no spectators. Discussions were audio-recorded. To identify citations each discussion was given an anonymized code. The discussion length was typically between 1 and 2 h. After transcription and anonymization, the analysis consisted of a sequence of paraphrasing, generalization, reduction, and summation (8, 9):

First, the material was carefully read and segmented into meaning units. These units were then paraphrased to preserve the core message in simplified terms. In the next step, these paraphrases were generalized to a higher level of abstraction, making them applicable across different contexts. Through a process of reduction, redundant or less relevant content was condensed, while still maintaining the essence of the statements. These abstracted segments were subsequently summarized and grouped to form initial thematic categories. The category systems and themes were developed inductively from the data, rather than based on predefined codes, ensuring that they remained closely tied to the participants’ perspectives expressed in the group discussion (7).

The material was analyzed independently by two readers (MG, KW). Any discrepancies were discussed and resolved in discussion. Finally, an interdisciplinary research workshop, which included researchers with medical, psychological, and social science backgrounds, was convened to review, discuss, and interpret key qualitative passages (10). This triangulation of interdisciplinary perspectives contributed to the depth of the analysis. The group discussions were performed in German. On the selected sample material, we used forward and backward translation to ensure semantic appropriate English translations of the content. Participant feedback on the material was not obtained. The transcripts were available to participants upon request. The analysis was performed using Microsoft Excel (Redmont, Washington, United States). Conducting the analysis, the COREQ (COnsolidated criteria for REporting Qualitative research) checklist was applied (Supplementary Table 2) (11).

## Results

### Main categories and themes resulting from qualitative content analysis

In total *N* = 17/18 (94.4%) of the elective students participated in the group discussions. Using qualitative analysis of the transcripts, 285 relevant single statements of interviewees were identified within the structured focus group discussions. Mean discussion duration was 45 min.

Regarding the category systems formed for Lugano course assessments, both common (Table 1) and location-specific (Table 2) categories emerged. Categories could be formed for “Organization and educational environment,” “Learning in the elective subject” and “Effects on the choice of continuing education.”

**TABLE 1:** Common categories and themes derived from the student group discussions.

**Organization and Educational Environment**
Teaching of advanced course content
No interfering courses
Feeling of appreciation
Good organization
Friendly and motivated personnel
**Learning in the Elective Subject**
Small groups promote learning
Desire for more practical learning
Group dynamics experienced as beneficial
Learning from a case
Learning through interaction
Learning on patients
Learning in functional areas
Education in Switzerland
Useful cardiology knowledge
Possible postgraduate training in cardiology
Further training/clerkships in cardiology planned
No change in postgraduate training desire
Encouragement in the desire to become a cardiologist
Concerns regarding intensive care medicine
Team and working environment important

**TABLE 2:** Location specific categories derived from the student group discussions.

**Comparison of the German and Swiss healthcare systems**
More resources
Less stressful working environment
More innovation
Information about further training/postgraduate training
Flat hierarchies/Non-hierarchial system
More attentive patient care
Problems with further training/postgraduate training
**Language Barrier**
Linguistic challenge perceived as appealing
Little prior contact with studying in English
Physicians speak good English; nursing staff only Italian
Staff go to great lengths to translate and minimize linguistic hurdles
Linguistic hurdles perceived as manageable
**Motivation**
More intensive involvement with cardiac content
Desire to attend more optional cardiology seminars
Desire for more cardiology teaching in the curriculum
Desire to propagate knowledge in the future similar to the way it was done in Switzerland
Exchange in the group is perceived as motivating

In the following, we provide summaries of the identified themes belonging to the main categories. Supporting anchor quotations are in Supplementary Table 3.

### Organization and educational environment

Students participating in the international electives were satisfied. They praised the broad content of the various modules, as well as the friendly and collegial atmosphere. For example, students reported that “*every staff member that you encountered was always friendly, nice and open-minded, you could always ask your questions, no matter what question.*”

Students stated that the absence of other courses had a positive effect. *“For me personally, the intensive elective course was better because I could just concentrate fully on it. You get out of your normal university courses, [.] I was to concentrate entirely on cardio again, I found it better for my learning process.”*, indicated how they were better able to adapt to the elective content.

### Learning processes in the elective subject

Regarding the learning experience, students “…*remember most about*…” learning from the professional discourse with clinically active lecturers and *“practical* [cases and patients]*, where we actually went down to the cardiac catheterization lab or to the intensive care unit or went to the cardiology congress and so forth*…”

As a critique point *(“which wasn’t so good”)*, the students *“might have expected a bit more of”* practical, clinically oriented learning. For example, they *“would have* [opted for] *more patient contact.”*

A participating student however, *“would recommend this course to anyone”* as he particularly *“found very good that the group size was so small* [providing] *the opportunity* [of more intensive teaching through] *one-on-one teaching*, [which he] *had never really experienced that at university.”*

The students described that they regularly “[We] *sat together in the evening and then talked again about something we had seen* [reflecting on an experience] *with the patient today or just something like that,”* providing insight into the positive effects of the group dynamics created by the block course that *“that somehow made it* [their experience] *nice.”*

### Impact on later professional life

Students *“found* [the elective] *very educational”* and reported to have gained *“a lot of interest* [in cardiology as a subject],” acquired useful specialized knowledge for their future careers and “*took an incredible amount away* […]*, especially the motivation.”* stating the electives’ learning experience “*was really good.”* Consequently, students also reflected on possible effects of the electives on their future careers. While some students indicated no change in their desired further training, others increasingly thought about *“whether I might go into cardiology.”*

A student directly indicated that the elective *“strengthened my desire to do cardiology, there’s no other way to put it.”*

The elective was also described as an aid in deciding to do postgraduate training or part of the required last stage of medical training, i.e., a portion of their last practical phase (PJ), in Switzerland: *“I had already applied for my PJ in Switzerland and had been thinking about it: Am I really going to do it or not, I’ve been putting off signing this contract the whole time, because then it’s so certain that you’re going away for 4 months and if you don’t do it, you have a contractual penalty and so on. Just to have the opportunity to be a fly on the wall, in theory to see how it works in Switzerland, to ask people and I asked a lot of people what it’s like in general and also critically questioned them.”*

### The elective in the context of the curriculum

The students independently evaluated their elective within the context of the local curriculum. Gauged against the wide range of internal medicine subjects, the students described a general dearth of cardiology teaching units in the clinical curriculum. In this respect, the cardiology elective was seen as a means of repeating and deepening previously acquired knowledge and as an opportunity to close knowledge gaps. The students described how certain content was made clear because of the elective: *“and I only really understood a lot of the connections, also through my presentation on heart failure, so there’s just such a plethora of material during the semester and firstly, so much is left out.”*

Emphasized as particularly positive were the practical, clinical aspects of the elective, which had high value as compared to their usual studies. In discussion *16_P5*, for example, the students described how the elective made them realize “…*where the focus is, what really happens in the clinic. From the book or from the lecture you only learn what there is or the rarest diseases, and here you learn about the “bread and butter” cases and what happens there. I found these impressions quite good. [*…*].“*

Students rated the elective as very efficient in terms of knowledge transfer compared to other courses such as medical clerkships. Ultimately, they perceived that they “…*were quite a bit ahead of our fellow students*…,” with regard “…*to studying, [*…*]* [for the] *internal medicine exam*….” They reflected their perceived learning success in the elective in relation to their lecture material: *“when you study [*…*] the lecture slides, it was always like this: Oh well, yes, you saw that, oh well, that was that and that - oh well, yes, exactly, uh, you still have it in your head, so it was quite relaxed to study now too.”*

### Comparison of the German and Swiss healthcare systems

The students reported in detail on the differences they had experienced in the healthcare system (Table 2).

The students described that “…*what* [they] *saw in Switzerland was simply a bit different in terms of the financial level and the personnel level than what we are used to* [in the German healthcare system].” and elaborated on more technical resources compared to home. They implied that “…*it just remains in the memory that cardiology can be done differently or internal medicine in general.”*

They also perceived a less stressful working environment and differences in the duration and intensity of the workday. *“you saw a lot from a different work culture. They have employment contracts for 55 h and I think they are there more than the 55 h, but you still had the feeling that they are always more relaxed, so they simply work, they don’t go in and work straight through, but also take a break, drink coffee, have a nice chat, that was my impression, I thought that was really good.”*

The students also perceived that they “*didn’t have the feeling that there was such a big hierarchy here* [in Lugano].” between resident physicians and attending or chief physicians. Compared to Germany, a more attentive and friendly approach to patients was noted. However, it was recognized that problems existed in the training of resident physicians in the form of a lack of rotations for all residents.

### The challenge of the language barrier

The language barrier between the German students and the Italian-speaking staff in Switzerland was another strong theme in the discussions, as they had little contact with foreign languages during their medical studies (Table 2).

Most of the communication with physicians took place in English; however, the majority of the communication with nursing personnel was only possible in Italian. Overall, the language barriers were described as manageable. In discussion *L16_P3 lines 74–78*, for example, the student described that *“conferences when they were in Italian, for example, were immediately stopped and then continued in English so that we could understand everything.”* The students acknowledged their host efforts of inclusion and that “…*even when it was rather more difficult for some doctors to speak in English, they still made an effort, so in any case they always switched immediately.”*

Finally, the students noted that *“the other language”* was an appealing linguistic challenge, that *“[.] definitely made it totally interesting for me was, the fact that you really deal intensively with medicine in English.”*

### Motivation

Among the elective participants, a particularly high level of motivation was revealed as reflected in the subcategories found in Table 2.

After course completing, the students repeatedly expressed that “[the elective] *totally motivated me in the sense that [.] I would*…” engage more intensively with cardiology teaching content for example “…*do another ECG course offered here by the university over 2 days because I said okay, now I’ve just got some basic knowledge.”*

A further motivating factor was the close exchange on cardiology topics in the elective group, which was enhanced by the physical proximity of the students.

Likewise, the students also took the type of knowledge and skills transfer they experienced as a role model. They found the teachers’ involvement was “*nice for a student, so you go out there with a completely different feeling and maybe have more fun with the subject.”* The students declared they “…*would make an effort later [.]”* to take similarly good care of medical students during their residency. They found that: *“[.] once you’ve experienced it like that and have gotten such a big benefit, then I hope that I personally can perhaps also take more care of the students during my residency.”*

## Discussion

We assessed the perceptions and learning experiences of medical students from Otto-von-Guericke-University, Magdeburg, Germany participating in an international cardiology elective in the Cardiocentro Ticino, Lugano, Switzerland. Analysis of qualitative focus groups revealed that an intensive course program had specific benefits: Regarding teaching and learning methods, there prevailed a consensus among the participants of the electives that teaching practical clinical and patient centered issues with high degree of interaction between the students and lecturers was desired. Particularly, the implicit elucidation of differences in the organization of the different healthcare systems and hospitals was noted. Furthermore, it was challenging for the German students in Lugano to experience medicine not only in a foreign context but also with different languages (English/Italian). A limitation mentioned by the participants was that, compared to their usual teaching at home, the interprofessional aspects during their time at the Cardiocentro Lugano were less comprehensible.

Ultimately, the elective students showed a high level of reflection of the issues provided and gained more motivation to continue studying cardiology as well as a strong tendency to consider cardiology as a specialist discipline.

In medical education, clinical clerkships abroad and the intensive examination of theoretical and practical training against a “foreign backdrop” have been increasingly studied and frequently used extracurricularly (12). In this context, effects such as improvement in foreign language skills, individual personal development, and the acquisition of social and intercultural skills are described (13). The impressions and experiences gained by students during the practical exercise of their theoretical skills often endure far into their professional lives. Fostering this peer-group learning significantly contributes to academic, social, and emotional development. It creates an active, collaborative learning environment that positively impacts cognitive growth, motivation, and group learning dynamics (14).

Thus, international undergraduate teaching collaborations may interest medical societies and disciplines beyond the expected benefits of training abroad, as they could reveal factors influencing students’ career choices (15). While traditional ERASMUS programs and university partnerships serve as institutional frameworks to promote international electives, they usually operate independently of clinical departments or those connected through research collaborations. As a result, they may or may not fully align with the practical training needs of medical students. Personal academic networks and those provided by medical societies, may offer additional targeted and discipline-specific opportunities for students to gain hands-on experience or connect students with international groups for thesis projects fostering collaboration in early career phases.

In a previous study, we found that without defined curricular objectives, medical clerkship students set their own goals, prioritizing practical skills and gaining clinical insights, shaped by social factors and performance insecurities (16). In contrast, the absence of social learning interaction may negatively impact perceived personal learning success (17). Participating students in the specialized, condensed cardiology elective prioritized understanding subject-specific processes and systematically advanced their knowledge. Contrary to previous findings (18), students in the Lugano course described that the language differences did not pose particular problems for their perceived learning effort.

Condensed electives abroad may offer unique advantages for participants, including exposure to different medical insights and increased enthusiasm for the subject, while also highlighting potential challenges in cross-border education. Many departments use small-group electives to promote their specialties, giving students the freedom to explore areas for further training. With the concept proposed in the present study, learners who prefer a compact elective may emerge as a particularly promising group for sustainable training formats that foster enthusiasm for the subject and may influence future specialty choices (2). In this way, the educational atmosphere and surrounding is perceived positively by the participants and seems to contribute to a favorable learning effect without pressure to perform. International short-term programs of 7–10 days, combining practical phases (comparable to clinical clerkships) with structured theoretical guidance, allow for intensive, focused learning within a clearly defined timeframe. The compact structure promotes focused attention and offers an ideal opportunity to gain deeper insights into specific topics (1).

Although this elective was conducted in Switzerland, its essential pedagogical components such as small-group learning, early specialty exposure with defined objectives, and structured reflection are not bound to local resources and as such may be transferable to other various educational contexts.

The format’s brevity and collaborative structure support adaptation to requirements with limited resources. Comparable programs could be implemented through academic partnerships, integration of digital teaching formats, and mentorship-based models. With clearly defined learning objectives and committed educators, effective international learning experiences may be feasible even under constrained conditions (19). Reported student benefits, such as enhanced motivation and interest, suggest a high degree of transferability when similar educational frameworks are employed. Focused international electives may therefore be meaningful and valuable as unique and learning opportunities (20).

Regarding our course, presumably, not just an interest in cardiology motivated the study abroad participants to register for the elective in Lugano. Both, the combination of the subject and the location were appealing, and students found their stay abroad very intensive in various aspects: Differences in the healthcare systems and clinical procedures encouraged criticism and reflection on the subject and learning content. International cooperation may so broaden horizons and encourage critical questioning (21). Students used the study abroad option to assess whether advanced training in Switzerland was desirable, gaining insight into local structures, processes, and conditions during the week-long elective (22). Ultimately, participating in the cardiology short-term elective deepened the students’ interest in the subject. Students in Lugano consistently reflected on their daily experiences throughout their elective, which is a crucial aspect of the learning process (23).

We suggest that well-structured electives, including study abroad opportunities, may serve as valuable components of the internal medicine curriculum and may be adaptable to other disciplines and settings. Additionally, fostering reflection and encouraging activities that push students beyond their comfort zones should be prioritized more often.

The cardiology clinic at the Otto-von-Guericke-University Magdeburg has created a broad range of elective courses and has reached interested parties through these courses. The student’s subjective experience of enthusiasm for and commitment to the subject may have a positive effect on the later specialty choices of the elective participants (2). These offers are therefore used to gain experience in medicine in a way that is geared toward student inclination. Furthermore, experience gained on the hospital wards is incorporated into this decision. Small group electives therefore appear to be excellent methods for exposing interested students to specialty areas and for giving them more tailored methods to develop in their potential field of choice.

One of the major strengths of the qualitative assessment is the open approach that enables the formation of new hypothesis and models (24). As a potential limitation, we acknowledge the small number of course participants, which however, was very accessible for a qualitative research approach (24), given we achieved content saturation in the discussion material. Moreover, qualitative research focuses on depth and insight, not generalizability (25). As such however, the results of the focus group discussions were entirely subjective, and we cannot exclude Asch’s effect influencing the group dynamic (26). Selection of students willing to travel abroad in the first place may have introduced a selection bias, and participant characteristics may belong to the same group of students who share similar opinions may potentially restrict the conclusions drawn.

In times of increasing resource scarcity, innovative approaches such as the one presented here are valuable for professional societies in their efforts to attract young talents. However, further studies, such as comparative research, are needed to validate and better contextualize the findings.

This is to gain additional knowledge to advance skills and improve upon education and training in cardiology (27). We therefore suggest that further investigations be carried out on international student courses to evaluate if indications of the present study may apply toward improvement of curricular teaching.

## Conclusion

The addition of specialized electives, especially study abroad electives offered in in a short time frame, to the medical school curriculum may have beneficial effects on students’ learning and help them to discern a specialty interest. Intensive medical education and learning experiences abroad may also influence the choice of location for future advanced training. These educational methods already exist at later stages of specialty training, consideration to implement such structures at an earlier juncture may provide additional patronage and support for students interested in the subject.

## Data Availability

The original contributions presented in this study are included in this article/Supplementary material, further inquiries can be directed to the corresponding author.

## References

[1] Stieger P Schildberg C Gottschalk M Werwick K Hunger J Walcher F Innovative fakultative seminarkonzepte besonders klinisch-praktisch ausgerichteter lehre zur famulatur- und PJ-vorbereitung aus spezifisch chirurgischer sicht. *Die Chir.* (2023) 94:432–40. 10.1007/s00104-022-01757-x 36418573 PMC10156815

[2] Werwick K Spura A Gottschalk M Meyer F Walcher F König S Für chirurgie begeistern? Einflüsse der famulatur aus sicht studierender auf eine spätere fachpräferenz. *Zentralbl Chir.* (2017) 142:550–9. 10.1055/s-0043-114732 28905346

[3] Gartmeier M Reimer M Huber J Epstein N Fischer MR Berberat PO. International mobility of students in the medical disciplines from a comparative perspective. *GMS J Med Educ.* (2020) 37:Doc34. 10.3205/zma001327 32566736 PMC7291386

[4] Petersdotter L Niehoff E Freund PA. International experience makes a difference: Effects of studying abroad on students’ self-efficacy. *Pers Individ Differ.* (2017) 107:174–8. 10.1016/j.paid.2016.11.040

[5] Brown M Boateng EA Evans C. Should I stay or should I go? A systematic review of factors that influence healthcare students’ decisions around study abroad programmes. *Nurse Educ Today.* (2016) 39:63–71. 10.1016/j.nedt.2015.12.024 27006034

[6] Hayashi M Son D Nanishi K Eto M. Long-term contribution of international electives for medical students to professional identity formation: A qualitative study. *BMJ Open.* (2020) 10:e039944. 10.1136/bmjopen-2020-039944 32801209 PMC7430439

[7] Mayring P. *Qualitative inhaltsanalyse. Grundlagen und techniken.* 10th ed. Basel: Beltz Verlag (2008).

[8] Dresing T Pehl T. *Praxisbuch interview, transkription & analyse. Anleitungen und regelsysteme für qualitativ forschende.* 6th ed. Marburg: Dresing (2015).

[9] Przyborski A Wohlrab-Sahr M. *Qualitative sozialforschung, ein arbeitsbuch.* 4th ed. München: Oldenbourg (2014). 10.1524/9783486719550

[10] Allert T Dausien B Mey G Reichertz J Riemann G. Forschungswerkstätten — programme, potenziale, probleme, perspektiven. In: Mey G Mruck K editors. *Qualitative forschung, analysen und diskussionen.* Berlin: Berliner Methodentreffen (2014). p. 291–316. 10.1007/978-3-658-05538-7_15

[11] Tong A Sainsbury P Craig J. Consolidated criteria for reporting qualitative research (COREQ): A 32-item checklist for interviews and focus groups. *Int J Qual Health C.* (2007) 19:349–57. 10.1093/intqhc/mzm042 17872937

[12] Huhn D Huber J Ippen FM Eckart W Junne F Zipfel S International medical students’ expectations and worries at the beginning of their medical education: A qualitative focus group study. *BMC Méd Educ.* (2016) 16:33. 10.1186/s12909-016-0549-9 26817850 PMC4730783

[13] Żebryk P Przymuszała P Nowak JK Cerbin-Koczorowska M Marciniak R Cameron H. The impact of ERASMUS exchanges on the professional and personal development of medical students. *Int J Environ Res Public Heal.* (2021) 18:13312. 10.3390/ijerph182413312 34948920 PMC8706907

[14] Zhang Y Maconochie M. A meta-analysis of peer-assisted learning on examination performance in clinical knowledge and skills education. *BMC Méd Educ.* (2022) 22:147. 10.1186/s12909-022-03183-3 35248051 PMC8897892

[15] Cleland JA Johnston PW Anthony M Khan N Scott NW. A survey of factors influencing career preference in new-entrant and exiting medical students from four UK medical schools. *BMC Med Educ.* (2014) 14:151. 10.1186/1472-6920-14-151 25056270 PMC4131477

[16] Gottschalk M Albert C Werwick K Spura A Braun-Dullaeus RC Stieger P. Students’ perception and learning experience in the first medical clerkship. *BMC Med Educ.* (2022) 22:694. 10.1186/s12909-022-03754-4 36167525 PMC9513910

[17] Gottschalk M Milch PM Albert C Werwick K Braun-Dullaeus RC Stieger P. Medical education during the Covid-19 pandemic long-term experiences of German clinical medical students. *PLoS One.* (2023) 18:e0286642. 10.1371/journal.pone.0286642 37279236 PMC10243622

[18] Takehara S Wright F Kawaguchi Y Ishida Y Morio I Tagami J. Characteristics of undergraduate dental students in Japan: English competency and willingness to study abroad. *Int Dent J.* (2016) 66:311–7. 10.1111/idj.12244 27283476 PMC9376638

[19] Henry JA Groen RS Price RR Nwomeh BC Kingham TP Hardy MA The benefits of international rotations to resource-limited settings for U.S. surgery residents. *Surgery.* (2013) 153:445–54. 10.1016/j.surg.2012.10.018 23274099

[20] Thompson MJ Huntington MK Hunt DD Pinsky LE Brodie JJ. Educational effects of international health electives on U.S. and Canadian medical students and residents. *Acad Med.* (2003) 78:342–7. 10.1097/00001888-200303000-00023 12634222

[21] Fabris E Kennedy MW Bode C. International subspecialty fellowship training, the path for cardiologists of tomorrow? A European perspective. *J Am Coll Cardiol.* (2017) 69:1200–3. 10.1016/j.jacc.2017.01.021 28254183

[22] Cherniak WA Drain PK Brewer TF. Educational objectives for international medical electives. *Acad Med.* (2013) 88:1778–81. 10.1097/acm.0b013e3182a6a7ce 24072105 PMC4019073

[23] Mordang SBR Vanassche E Smeenk FWJM Stassen LPS Könings KD. Residents’ identification of learning moments and subsequent reflection: Impact of peers, supervisors, and patients. *BMC Med Educ.* (2020) 20:484. 10.1186/s12909-020-02397-7 33267810 PMC7709399

[24] Flick U von Kardorff E Keupp R von Rosenstiel L Wolff S. *Qualitative forschung: Ein handbuch.* 11th ed. Reinbek bei Hamburg: Rowohlt Taschenbuch Verlag (2015).

[25] Leung L. Validity, reliability, and generalizability in qualitative research. *J Fam Med Prim Care.* (2015) 4:324–7. 10.4103/2249-4863.161306 26288766 PMC4535087

[26] Asch SE. Effects of group pressure on the modification and distortion of judgements. In: Guetzknow H editor. *Groups, leadership and men.* Pittsburgh, PA: Carnegie Press (1951). p. 177–90.

[27] McDonagh TA Gardner RS Lainscak M Nielsen OW Parissis J Filippatos G Heart failure association of the European society of cardiology specialist heart failure curriculum. *Eur J Hear Fail.* (2014) 16:151–62. 10.1002/ejhf.41 24464608

