# Supplementary material for: Medical education abroad: experience and perceived learning effects of German medical students in an international cardiology elective
Source: Front Med (Lausanne). 2025 Jul 9;12:1556761. doi: 10.3389/fmed.2025.1556761 (PMC12283788; doi:10.3389/fmed.2025.1556761)
Supplement: Supplementary file 1 [file Data_Sheet_1.pdf]

German medical students' educational experience in a cardiology elective in Switzerland versus Germany: A qualitative analysis

Supplemental Table 1. Interview Guideline in German and English translation.

**Leitfaden für Gruppendiskussion (German)**	
1	Was waren Ihre Gründe und Motivation zur Auswahl des Wahlfaches Kardiologie (Lugano vs. Magdeburg)?
2	Was waren Ihre Erwartungen an das Wahlfach Kardiologie?
3	Welche Erwartungen sind erfüllt worden?
4	Warum haben Sie sich für das Wahlfach im Ausland entschieden?
5	Was sind die Vorzüge und Nachteile eines Wahlfaches in Lugano/in Deutschland?
6	Was hat Ihnen besonders gefallen und aus welchem Grund?
7	Was hat Ihnen nicht gefallen und was könnte geändert werden?
8	Was sind die wichtigsten Erfahrungen, die Sie aus diesem Wahlfach mitgenommen haben?
9	Hatte Ihre Wahlfachteilnahme Einfluss auf die spätere Berufswahl und wenn ja in welcher Form?
10	Welchen Effekt (Chancen/Limitationen) hat aus Ihrer Sicht eine internationale Lehrkooperation?
11	Würden Sie das Wahlfach ihren Kommilitonen weiterempfehlen?

**Interview Guideline for the Group Discussions (English)**	
1	What were your reasons and motivation for choosing the cardiology elective (Lugano vs. Magdeburg)?
2	What were your expectations regarding the cardiology elective?
3	Which expectations were met?
4	Why did you choose to participate in the elective abroad?
5	What are the advantages and disadvantages of the elective in Lugano/Germany?
6	What did you particularly like and for what reason?
7	What did you not like and what could be changed?
8	What are the most important experiences you took away from your elective participation?
9	Did your elective participation influence your later career choice and if so, in what way?
10	From your perspective, what effect (opportunities/limitations) does international teaching cooperation have?
11	Would you recommend the cardiology elective to your fellow students?
