# Supplementary material for: Medical education abroad: experience and perceived learning effects of German medical students in an international cardiology elective
Source: Front Med (Lausanne). 2025 Jul 9;12:1556761. doi: 10.3389/fmed.2025.1556761 (PMC12283788; doi:10.3389/fmed.2025.1556761)
Supplement: Supplementary file 3 [file Data_Sheet_3.pdf]

Supplemental Table 3. Categories, themes and anchor quotations derived from the Lugano elective

**Lugano Elective**		**Wahlfach Lugano**	
**Organization and Educational Environment**		**Organisation und Lernatmosphäre**	
Friendly and motivated personnel	"[...] every staff member that you encountered was always friendly, nice and open-minded, you could always ask your questions, no matter what question [...]" Lug15_P1 Lines 112-116	Freundliches und motiviertes Personal	„[…] jeder Mitarbeiter den man angetroffen hat war immer freundlich, nett und aufgeschlossen, man konnte immer seine Fragen stellen, egal welche Frage […]" Lug15_P1 Zeile 112-116
Feeling of appreciation	"Yes, you just have the feeling that you are totally valued as a student, so ...you often have that feeling often in the elective subjects here, but rarely in the regular courses and um, I found that really powerful, [...]" Lug15_P4 Lines 123-128	Gefühl der Wertschätzung	"Ja man hat halt das Gefühl man wird total wertgeschätzt als Student, also ...das Gefühl hatman in den Wahlfächern auch hier oft, aber in den regulären Kursen halt selten und ähm, das fand ich halt stark, […]" Lug15_P4 Zeile 123-128
Good Organization	" [...] um, organization is really great [...]" Lug16_P4 Line 116	Gute Organisation	" […] ähm, Organisation echt großartig […]" Lug16_P4 Zeile 116
Teaching of advanced course content	"For example, I was in the cardiac catheterization lab, I was standing at the table, and then I was really dressed in sterile clothes and then it was like: 'Yes here, feel the pulse here and uh have you ever placed an arterial catheter?' And I was like: Not yet on a patient but I've seen it many times but not done it yet, and he was like: 'Well, the principle is actually the same now, it's just....' and then he just explained and said: 'Here take a look, feel it '[...]" Lug15_P1 Lines 354-360	Vermittlung von weiterführenden Lehrinhalten	„Ich war zum Beispiel im Herzkatheterlabor, stand ich mit am Tisch und dann war ich auch wirklich so auch Steril mit eingekleidet und dann hieß es so :Ja hier, fühl mal hier den Puls ne hm und äh Hast du schonmal eine Arterie gelegt? Und ich so: Noch nicht am Patienten aber ich habs schon oft gesehen aber noch nicht gemacht und er so : Naja, das Prinzip ist jetzt eigentlich das gleiche , liegt halt....und dann hat er halt erklärt und hat immer gesagt :Hier guck mal, taste mal […]" Lug15_P1 Zeile 354-360
No interfering courses	"For me personally, the intensive elective course was better because I could just concentrate fully on it. You get out of your normal university courses, [...] yes, for me, I was to concentrate entirely on cardio again, I found it better for my learning process [...]" Lug17_P2 Lines 171-178	Keine interferierenden Lehrveranstaltungen	„Also für mich persönlich war der Intensivwahlkurs besser, weil ich mich einfach voll und ganz drauf konzentrieren konnte. Man kommt aus seinem normalen Unikursen raus, hat den Kopf vielleicht noch voll, schreibt Klausuren, hat irgendwie noch Vorlesungen oder so […] ja. also für mich persönlich war das sich ganz auf Kardio nochmal zu konzentrieren, fand ich für meinen Lernprozess besser […]" Lug17_P2 Zeile 171-178

Learning Processes in the Elective Subject		Lernprozesse im Wahlfach	
Learning from a case	"[...] and then on the same day we went down to the operating room, so that was cool for us. They just took us by the hand and did the indications with us and not like this: We'll show you how it's done, but rather: Here you take the template and now tell me whether, uh, the ECG is appropriate, whether it fits, whether we can do it and then we sort of made the indication together with them [...]" Lug15_P2 Lines 331-337	Lernen am Fall	„[…] und der ist dann halt am gleichen Tag dann noch runter in den OP gefahren also das war cool für uns. Haben die uns einfach an die Hand genommen und haben mit uns die Indikationsstellung gemacht und jetzt nicht so: Wir zeigen euch jetzt mal wies geht sondern: Hier nehmt ihr die Schablone und jetzt sag mir mal ob äh, das EKG angebracht ist, ob das so passt, ob wir das machen können und dann haben wir mit denen zusammen dann quasi die Indikation gestellt […]“ Lug15_P2 Zeile 331-337
Learning on patients	"[...] now in ultrasound, for example, that this is then shown on one patient and the next patient is then asked: What then is this now? Or what is that? And is what we have just measured normal or not? And so you get a lot of explanations first [...]" Lug15_P1 Lines 149-153	Lernen am Patienten	“[…] jetzt im Ultraschall zum Beispiel dass das dann halt an einem Patient gezeigt wird und beim nächsten Patient heißt es dann: Was ist den diss jetzt? Oder was ist das? Und ist denn das was wir da jetzt gemessen haben normal oder nicht? Und so das man halt viel erstmal erklärt bekommt […]“ Lug15_P1 Zeile 149-153
Learning in functional areas	"[...] So she put in a stent and did this balloon procedure, she just said yes here I'll show you how it is, touch here and then she just inflated it again so that you get a feeling for it again, these are things that you very rarely just get to see [...]" Lug15_P1 Lines 360-365	Lernen in Funktionsbereichen	„[…] also einen Stent gesetzt und so dieses Ballonverfahren gemacht hat se halt gesagt ja hier ich zeig dir jetzt mal wie das ist, fass mal hier an und dann hat se dann halt nochmal das aufgebläht damit man nochmal ein Gefühl dafür kriegt so das sind halt Sachen die kriegt man sehr selten einfach so mit […]“ Lug15_P1 Zeile 360-365
Learning through interaction	"[...] So this is really direct and that you are really asked to participate [...]" Lug16_P5 Lines 138-139	Lernen durch Interaktion	„[…] Also dieses wirklich direkte und das man auch wirklich aufgefordert wird mitzumachen […]“ Lug16_P5 Zeil1 138-139
Desire for more practical learning	"I would have liked a bit more clinical, we had already said that others would do a bit of an echo themselves, or I don't know, you were standing at the table. Or, ah well, you can't do much there either, but still just a little bit. It was quite a lot of talk, but not much so that you just do something yourself again, exactly." Lug15_P2 Lines 319-323	Wunsch nach mehr praktischem Lernen	„Ein bisschen mehr Klinik hätte ich mir gewünscht das hatten wir ja schon gesagt das andere ein bisschen nochmal selber ein Echo machen oder ich weiß nicht, standet ihr mit am Tisch? Oder ah ja gut, viel kann man da auch nicht machen aber trotzdem einfach noch ein bisschen, es war ziemlich viel Vortrag aber wenig, dass man da einfach nochmal selbst irgendwas macht, genau.“ Lug15_P2 Zeile 391-323
Group dynamics experienced as beneficial	"But I just think that somehow made it nice again, because I mean how often we sat together in the evening and then talked again about something we had seen or something [...] uh yes, uh with the patient today or just something like that [...]" Lug15_P1 Lines 238-243	Gruppendynamik als förderlich erlebt	“Aber ich find grade das hats halt irgendwie auch nochmal schön gemacht, weil ich meine wie oft saßen wir Abends zusammen und haben dann auch nochmal über irgendwas geredet oder was wir gesehen haben oder so […] äh ja... äh bei dem Patienten heute oder bei dem einfach so […]” Lug15_P1 Zeile 238-243

Small groups promote learning	"[...] what I found very good was that the group size was so small, I would recommend this course to anyone, I think it's good that we were only six people because then you had the opportunity to do this one-on-one teaching, I had never really experienced that at university [...]" Lug16_P5 Lines 135-138	Kleingruppen förderlich für Lernerfolg	“[…] was ich sehr gut fand ist das die Gruppengröße nur so klein ist, ich würds jedem weiterempfehlen diesen Kurs, ich finds gut, dass wir nur 6 Leute sind da man eben dann die Möglichkeit hat dieses One to one teaching zu machen das hab ich in der diesein der Uni hab ich das eigentlich noch nie erlebt […]” Lug16_P5 Zeile 135-138
**Comparison of the German and Swiss healthcare systems**		**Vergleich Gesundheitssystem Deutschland-Schweiz**	
More resources	"Well, it just remains in the memory that cardiology can be done differently or internal medicine in general, because what we saw in Switzerland was simply a bit different in terms of the financial level and the personnel level than what we are used to here [...]" Lug15_P3 Lines 74-79	Mehr Ressourcen	„Naja es bleibt halt einfach mal im Gedächtnis, dass man Kardiologie auch anders machen kann oder generell Innere Medizin, weil das wir in der Schweiz gesehen haben war ja vom finanziellen Niveau und vom personellen Niveau her einfach ein bisschen was anderes, als das war wir hier gewohnt sind […]“ Lug15_P3 Zeile 74-79
Less stressful working environment	"[...] you saw a lot from a different work culture. They have employment contracts for 55 hours and I think they are there more than the 55 hours, but you still had the feeling that they are always more relaxed, so they simply work, they don't go in and work straight through, but also take a break, drink coffee, have a nice chat, that was my impression, I thought that was really good." Lug15_P4 Lines 400-408	Weniger stressiges Arbeitsklima	„[…] hat man viel von einer anderen Arbeitskultur gesehen also die haben da Arbeitsverträge über 55 Stunden und die sind glaub ich auch eher mehr da als die 55 Stunden aber man hatte trotzdem das Gefühl die sind immer entspannter also die arbeiten halt, die gehen halt nicht rein und arbeiten straight durch sondern machen auch mal eine Pause, trinken Kaffee, unterhalten sich nett, so war mein Eindruck, das fand ich total gut.“ Lug15_P4 Zeile 400-408
More innovation	"So I have the feeling that they are always on the ball when it comes to addressing new developments and have also had some current people there who are somehow developing something new technically, especially when it comes to cardiac echo, for example, and HD echo, so imaging is strong there.strong, but otherwise I also had the feeling that they were always on the ball [...]" Lug15_P4 Lines 140-144.	Mehr Innovation	“Also das Gefühl die sind immer am Ball was so die aktuellen Entwicklungen angeht und haben auch irgendwie aktuelle Leute teilweise da gehabt, die irgendwie technisch was neues entwickeln, gerade auch das was Herzecho angeht zum Beispiel und HD Echo, also Bildgebung ist da halt Stark, aber auch ansonsten hatte ich das Gefühl, die bleiben immer am Ball […]“ Lug15_P4 Zeile 140-144.
Information about further training / postgraduate training	"[...] just to have the opportunity to play a little mouse in theory, how does it work in Switzerland, to ask people, and I asked a lot of people, also critically, how is it in general.For example, I was like with this 55/60 hour week and I thought, okay, maybe that's quite nice for a while, but I wouldn't want to spend so much time in the clinic for 20 years, I don't think. You could really ask them quite openly, they just answered quite openly [...]" Lug15_P1 Lines 604-610	Informationen über Weiterbildung	„[…] einfach so die Möglichkeit zu haben schonmal so Mäusschen zu spielen in der Theorie wie läuft das so in der Schweiz, auch die Leute halt zu fragen und ich hab halt auch viele denn so gefragt wie es denn so allgemein ist und halt auch kritisch hinterfragt, also ich war ja zum Beispiel dieser 55/60 Stunden Woche hab ich so gedacht, okay das is vielleicht ne zeitlang ganz nett, aber das würd ich keine 20 Jahre glaub ich wollen, so viel Zeit in der Klinik zu verbringen […]“ Lug15_P1 Zeile 604-610

Flat hierarchies / Non-hierarchial system	"Well, I didn't have the feeling that there was such a big hierarchy here [...]" Lug16_P1 Line 208	Flache Hierarchien	“Also ich hatte auch nicht das Gefühl, dass hier so eine große Hierarchie ist […]“ Lug16_P1 Zeile 208
More attentive patient care	"[...] I also felt that patient contact was much more intense, just the way we dealt with each other in the cardiac catheterization lab, I also noticed that the nurse addressed the patients on a first-name basis and um addressed everyone by their first names [...]" Lug15_P1 Lines 503-508	Aufmerksamerer Patientenumgang	„[…] also auch Patientenkontakt gefühlt viel intensiver, also schon alleine wie auch miteinander umgegangen war im Herzkatheterlabor ist mir das auch aufgefallen, dass die Schwester da die Patienten geduzt hat und ähm dann halt so alle mit Vornamen angesprochen […]“ Lug15_P1 Zeile 503-508
Problems with further training / postgraduate training	"Yes, the message was that a lot of things have really worked out for us now, which was of course nice for us, but that otherwise the training courses, for example within the clinic, unfortunately don't always work out so well for the residents [...]" Lug15_P1 Lines 105-110	Probleme mit Weiterbildung	“Ja, die Aussage war halt das jetzt für uns halt wirklich viel aufgegangen wurde, was für uns natürlich schön war, aber das halt sonst das mit den Fortbildungen zum Beispiel klinikintern gerade so für die Assistenzärzte halt leider nicht immer so gut klappt […]“ Lug15_P1 Zeile 105-110
**Language Barrier**		**Sprachbarriere**	
Linguistic challenge perceived as appealing	"[...] the second thing that definitely made it totally interesting for me was the other language, the fact that you really deal intensively with medicine in English [...]" Lug15_P6 lines 561-564	Sprachliche Herausforderung als reizvoll wahrgenommen	„[…] das zweite, was auf jeden Fall was es für mich auch total interessant gemacht hat war die andere Sprache, dass man auf Englisch halt wirklich intensiv sich mit der Medizin auseinandersetzt […]“ Lug15_P6 Zeile 561-564
Little prior contact with studying in English	"The situation of having to give a presentation in English was unusual for me. I had reservations, although I actually have no problem speaking in front of people. I realized that I was rushing through my sentences the first time during my presentation because it was a new situation for me." Lug15_P1 Lines 703-709	Bisher wenig Berührungspunkte mit Studium auf Englisch	„Die Situation einen Vortrag auf Englisch halten zu müssen war für mich ungewohnt. Ich hatte Berührungsängste, obwohl ich eigentlich kein Problem damit habe vor Leuten zu reden. Ich habe gemerkt, dass ich die erste Zeit bei meinem Vortrag durch meine Sätze durchgerast bin, weils für mich eine neue Situation war.“ Lug15_P1 Zeile 703-709
Physicians speak good English; nursing staff only Italian	"[...] the majority of the staff, including nursing personnel, really didn't speak English. The doctors did." Lug15_P3 Lines 393-395	Ärzte sprechen gutes Englisch; Pflegepersonal nur italienisch	„[…] der große Teil des Personals halt auch wirklich kein englisch gesprochen haben, also das Pflegepersonal, die Ärzte schon.“ Lug15_P3 Zeile 393-395
Staff go to great lengths to translate and minimize linguistic hurdles	"[...] conferences when they were in Italian, for example, were immediately stopped and then continued in English so that we could understand everything, even when it was rather more difficult for some doctors to speak in English, they still made an effort, so in any case they always switched immediately [...]" Lug16_P3 lines 74-78	Personal sehr bemüht, zu Übersetzen und sprachliche Hürden gering zu halten	„[…] Besprechungen als die zum Beispiel in Italienisch waren, wurde es sofort unterbrochen und in Englisch weitergeredet damit wir halt alles verstehen, auch wenn es für manchen für manche Ärzte eher schwieriger war auf Englisch zu reden haben sie sich doch Mühe gegeben, also auf jeden Fall haben sie immer sofort umgeswitcht […]“ Lug16_P3 Zeile 74-78
Linguistic hurdles perceived as manageable	“The English language made learning much more intense for me because I had to think more about what was exactly being said.” Lug15_P1 Lines 691-696	Sprachliche Hürden als bewältigbar wahrgenommen	Die englische Sprache hat das Lernen viel intensiver für mich gemacht, weil ich mehr darüber nachdenken musste, was da gerade gesagt wurde. Lug15_P1 Zeile 691-696

Motivation		Motivation	
More intensive involvement with cardiac content	"You pay more attention to cardiac findings such as edema, have a cardiologist's perspective, approach patients differently." Lug15_P2 Lines 65-73	Intensiveres Befassen mit kardiologischen Inhalten	„Man achtet mehr auf kardiologische Befunde wie z.B. Ödeme, hat einen kardiologischen Prüfblick, geht anders an Patienten heran.“ Lug15_P2 Zeile 65-73
Desire to attend more optional cardiology seminars	"It totally motivated me in the sense that I said I would do another ECG course offered here by the university over two days because I said okay, now I've just got some basic knowledge [...]" Lug15_P1 Lines 177-180	Wunsch mehr fakultative kardiologische Lehrveranstaltungen zu besuchen	„Mich hats insofern total motiviert das ich auch gesagt habe ich mach jetzt direkt nochmal einen EKG Kurs jetzt der hier von der Uni angeboten wird der über 2 Tage geht weil ich gesagt habe okay, jetzt hab ich gerade so ein Grundwissen […]“Lug15_P1 Zeile 177-180
Desire for more cardiology teaching in the curriculum	"I was already very interested in cardiology before and that has now been intensely confirmed once again, simply so that I now go to the lecture totally motivated and am actually already working towards it [...]" Lug15_P5 Lines 456-459	Mehr Lust auf curriculare kardiologische Lehre	„Ich war ja vorher schon sehr Kardio interessiert und das hat sich jetzt nochmal krass bestätigt, einfach so dass ich jetzt auch total motiviert in die Vorlesung gehe und darauf eigentlich schon zuarbeite […]“ Lug15_P5 Zeile 456-459
Desire to propagate knowledge in the future similar to the way it was done in Switzerland	"Yes, well, um, I find that once you've experienced it like that and yourself have gotten such a big benefit, then I hope that I personally can perhaps also take more care of the students during my residency. Of course it's a job with all the pressure from above, you have to do everyone justice somehow, but that's nice for a student, so you go out there with a completely different feeling and maybe have more fun with the subject and yes, I would make an effort later [...]" Lug17_P3 Lines 104-109	Wunsch Wissen selbst in Zukunft ähnlich gut zu vermitteln wie in der Schweiz	„Ja also ähm ich finde wenn man das einmal so erlebt hat und selbst da so einen großen Benefit rausgenommen hat , dann hoffe ich das ich persönlich dann vielleicht auch in meiner Assistenzarztzeit mehr so mich um die Studenten kümmern kann, klar es ist halt ein Job mit allem Druck von oben, man muss jeden irgendwie gerecht werden, aber das ist für einen Student schön, so geht da mit einem ganz anderen Gefühl raus und hat vielleicht auch mehr Spaß dann an dem Fach und ja da würd ich mir Mühe geben später […]“ Lug17_P3 Zeile 104-109
Exchange in the group is perceived as motivating	"[...] and I just had the feeling that we had grown together really well as a group, so to speak; Also in the evening nobody had to do anything or anything like that, but we were just there and that was the only thing that counted in these 5 days, that was nice. that was very nice [...]" Lug15_P4 Lines 205-209	Austausch in der Gruppe als motivierend empfunden	„[…] und ich hatte halt das Gefühl das wir als Gruppe sozusagen auch dann abends super zusammengewachsen waren und nicht jeder noch irgendwas erledigen musste oder so sondern wir einfach dort waren und das war dann halt das einzige was in diesen 5 Tagen gezählt hat, das war schön. das war sehr schön […]“ Lug15_P4 Zeile 205-209

	Impact on later professional life		Auswirkungen auf das spätere Berufsleben
Useful cardiology knowledge	"[...] I think I've learned a lot, especially the ECG course as has already been said, really great. I think we learn a lot by heart during our studies and understand very little [...]" Lug16_P5 Lines 114-116	Nützliches kardiologisches Fachwissen	"[…] ich glaub ich hab viel gelernt insbesondere den EKG Kurs wurde jetzt ja auch schon gesagt, ganz klasse ich find im Studium lernen wir viel auswendig und verstehen wenig […]" Lug16_P5 Zeile 114-116
No change in career aspirations	"Well, it hasn't changed for me either, I've already done a lot of cardiology before as I'd considered doing it in the past, but then I decided through various clinical traineeships that it wasn't for me, forever, but I think I want to be a bit fit and that's why I'd like to get involved with it.like to get involved with it." Lug17_P2 Lines 285-289	Keine Änderung des Weiterbildungswunsches	„Also für mich hat sichs auch nicht geändert, ich hab mich ja vorher auch schon viel ähm mit Kardio beschäftigt, hatte auch früher überlegt das mal zu machen aber hab dann auch durch diverse Famulaturen entschieden, dass es für mich nichts ist, für immer, aber ich finde, also ich für mich möchte da doch ein bisschen fit sein und deswegen möchte ich mich da gern mit beschäftigen.“ Lug17_P2 Zeile 285-289
Possible postgraduate training in cardiology	"[Different further training] I've now thrown that out after the elective, because I have said I will definitely do something with the heart. So that still hasn't been firmly fixed, but at least I can now imagine it much more easily." Lug17_P4 Lines 275-281	Kardiologie als mögliche Weiterbildung	„[Andere Weiterbildung] das hab ich jetzt rausgeschmissen nach dem Wahlfach, weil ich gesagt hab, ich mach jetzt auf jeden Fall glaub ich was mit Herz. Also das ist immer noch nicht festgelegt, aber zumindest kann ich mir das jetzt nochmal viel eher vorstellen.“ Lug17_P4 Zeile 275-281
Encouragement in the desire to become a cardiologist	"Yes, it also strengthened my desire to do cardiology, there's no other way to put it [...]" Lug15_P3 Lines 460-463	Bestärkung im Wunsch Kardiologe zu werden	„Ja mich hat das halt auch im Wunsch Kardiologie zu machen bestärkt, kann man nicht anders sagen […]“ Lug15_P3 460-463
Education in Switzerland	"I had already applied for my PJ in Switzerland and had been thinking about it: Am I really going to do it or not, I've been putting off signing this contract the whole time, because then it's so certain that you're going away for four months and if you don't do it, you have a contractual penalty and so on.Just to have the opportunity to be a fly on the wall, in theory to see how it works in Switzerland, to ask people and I asked a lot of people what it's like in general and also critically questioned them [...]" Lug15_P1 Lines 600-607	Ausbildung in der Schweiz	"Ich hatte mich ja schon für mein PJ in der Schweiz beworben und hatte halt überlegt: Mach ich das jetzt wirklich oder mach ichs nicht, habs noch die ganze Zeit so rausgezögert diesen Vertrag halt zu unterschreiben, weil es dann halt so gewiss ist das man für 4 Monate weggeht und wenn mans nicht macht, hat man ja auch Vertragsstrafe und so und einfach so die Möglichkeit zu haben schonmal so Mäuschen zu spielen in der Theorie wie läuft das so in der Schweiz, auch die Leute halt zu fragen und ich hab halt auch viele denn so gefragt wie es denn so allgemein ist und halt auch kritisch hinterfragt […]" Lug15_P1 Zeile 600-607
